# Distinct neurogenetic mechanisms establish the same chemosensory valence state at different life stages in *Caenorhabditis elegans*

**DOI:** 10.1093/g3journal/jkad271

**Published:** 2023-11-23

**Authors:** Navonil Banerjee, Elisa J Rojas Palato, Pei-Yin Shih, Paul W Sternberg, Elissa A Hallem

**Affiliations:** Department of Microbiology, Immunology, and Molecular Genetics, University of California, Los Angeles, CA 90095, USA; Molecular Biology Institute, University of California, Los Angeles, CA 90095, USA; Department of Microbiology, Immunology, and Molecular Genetics, University of California, Los Angeles, CA 90095, USA; Division of Biology and Biological Engineering, California Institute of Technology, Pasadena, CA 91125, USA; Department of Ecology, Evolution and Environmental Biology, Columbia University, NewYork, NY 10027, USA; Zuckerman Mind, Brain, Behavior Institute, Columbia University, NewYork, NY 10027, USA; Division of Biology and Biological Engineering, California Institute of Technology, Pasadena, CA 91125, USA; Department of Microbiology, Immunology, and Molecular Genetics, University of California, Los Angeles, CA 90095, USA; Molecular Biology Institute, University of California, Los Angeles, CA 90095, USA

**Keywords:** carbon dioxide response, *Caenorhabditis elegans*, chemosensory behavior, sensory valence, gas sensing, dauer larva

## Abstract

An animal's preference for many chemosensory cues remains constant despite dramatic changes in the animal's internal state. The mechanisms that maintain chemosensory preference across different physiological contexts remain poorly understood. We previously showed that distinct patterns of neural activity and motor output are evoked by carbon dioxide (CO_2_) in starved adults vs dauers of *Caenorhabditis elegans*, despite the two life stages displaying the same preference (attraction) for CO_2_. However, how the distinct CO_2_-evoked neural dynamics and motor patterns contribute to CO_2_ attraction at the two life stages remained unclear. Here, using a CO_2_ chemotaxis assay, we show that different interneurons are employed to drive CO_2_ attraction at the two life stages. We also investigate the molecular mechanisms that mediate CO_2_ attraction in dauers vs adults. We show that insulin signaling promotes CO_2_ attraction in dauers but not starved adults and that different combinations of neurotransmitters and neuropeptides are used for CO_2_ attraction at the two life stages. Our findings provide new insight into the distinct molecular and cellular mechanisms used by *C. elegans* at two different life stages to generate attractive behavioral responses to CO_2_.

## Introduction

Chemosensation is a critical sensory modality that contributes to locating food, finding mates or hosts, and avoiding pathogens and predators (Hildebrand and Shepherd 1997). In some cases, the valence of a chemosensory stimulus, i.e. whether the stimulus is attractive or aversive, depends on an animal's physiological state (Ribeiro and Dickson 2010; Wasserman *et al.* 2012; Wasserman *et al.* 2013; Devineni and Scaplen 2021). However, in other cases, chemosensory valence remains constant despite dramatic changes in internal physiology (Schmidt and Beauchamp 1988; Hansson *et al.* 2010; Oleszkiewicz *et al.* 2022; Banerjee *et al.* 2023). The molecular and cellular mechanisms that enable animals to maintain the same valence state across widely varying physiological conditions remain poorly understood.

The response of the free-living nematode *Caenorhabditis elegans* to carbon dioxide (CO_2_) offers a powerful model system for exploring the mechanisms that determine chemosensory valence (Banerjee and Hallem 2020). While well-fed adults are repelled by CO_2_, starved adults are attracted to CO_2_ (Bretscher *et al.* 2008; Hallem and Sternberg 2008; Bretscher *et al.* 2011; Hallem, Spencer, *et al.* 2011; Rengarajan *et al.* 2019). In addition, dauer larvae—long-lived, nonfeeding, stress-resistant larvae that form in response to adverse environmental conditions (Hu 2007)—are attracted to CO_2_ (Hallem, Dillman, *et al.* 2011a; Banerjee *et al.* 2023). Thus, a comparison of the molecular and cellular mechanisms that drive CO_2_ attraction in dauers vs starved adults can provide insight into how different life stages establish similar chemosensory preferences.

We previously showed that although both starved adults and dauers are attracted to CO_2_, the neural activity dynamics and the motor outputs evoked by CO_2_ differ across the two life stages (Rengarajan *et al.* 2019; Banerjee *et al.* 2023). For example, the AIY, RIG, and AIB interneurons show different CO_2_-evoked calcium responses in starved adults vs dauers. AIY displays stochastic excitatory CO_2_-evoked activity in starved adults (Rengarajan *et al.* 2019) but inhibitory CO_2_-evoked activity in dauers (Banerjee *et al.* 2023), whereas RIG and AIB do not respond to CO_2_ in starved adults but display excitatory CO_2_-evoked activity in dauers (Rengarajan *et al.* 2019; Banerjee *et al.* 2023). However, how these distinct CO_2_-evoked patterns of neural activity contribute to CO_2_ attraction was unclear.

In this study, we correlate the CO_2_-evoked activities of these interneurons with their roles in promoting CO_2_ attraction in starved adults vs dauers. We show that individual interneurons make distinct contributions to CO_2_ attraction at the two life stages. Whereas AIY promotes CO_2_ attraction in starved adults but not dauers, RIG and AIB promote CO_2_ attraction in dauers but not starved adults. We also show that insulin signaling, which promotes CO_2_-evoked AIB activity in dauers (Banerjee *et al.* 2023), functions in neurons to selectively drive CO_2_ attraction in dauers. In addition, we identify distinct combinatorial codes of neurotransmitters and neuropeptides that promote CO_2_ attraction at the two life stages. Our results illuminate the different neurogenetic mechanisms that operate in dauers vs adults to establish the same chemosensory valence state.

## Materials and methods

### *C. elegans* strains

Worms were cultured on 2% nematode growth media (NGM) plates seeded with *Escherichia coli*OP50 bacteria at ambient temperature (∼22°C) and CO_2_ (∼0.038%) as previously described (Stiernagle 2006; Scott 2011; Banerjee *et al.* 2023). The temperature-sensitive strains CB1370, DR1565, JT191, EAH404, EAH407, and EAH408 were maintained at 15°C but were moved to ambient temperature (∼22°C) at least 24 h prior to experiments to minimize any effects of temperature shifts on behavior, as previously described (Banerjee *et al.* 2023). The strains JT709 and BQ1 were treated similarly for direct comparison with strain DR1565 in Fig. 2f. A complete strain list is provided in Supplementary Table 1.

### Molecular biology and transgenesis

The strains where specific neurons were genetically ablated or silenced were generated in previous studies (Kunitomo *et al.* 2013; Luo *et al.* 2014; Calhoun *et al.* 2015; Jin *et al.* 2016; Guillermin *et al.* 2017). The *inx-6*^*AIB OFF*^ strain, where *inx-6* function was eliminated specifically in AIB, was generated in a previous study (Bhattacharya *et al.* 2019). For tissue-specific rescue of *daf-2*, the following plasmids containing the *daf-2* cDNA were obtained from Addgene and individually injected into the CB1370*daf-2(e1370)* strain at 30 ng/µL, along with P*myo-2*::*dsRed* (20 ng/µL) as a coinjection marker and pBlueScript (50 ng/µL): pJH4531 (P*rgef-1*::*daf-2*) for pan-neuronal rescue (Addgene #132366), pJH4723 (P*ges-1*::*daf-2*) for intestinal rescue (Addgene #178899), and pJH4733 (P*myo-3*::*daf-2*) for muscle rescue (Addgene #178898) (Hung *et al.* 2014).

### CO_2_ chemotaxis assays

CO_2_ chemotaxis assays on starved adults and dauers were performed as previously described (Rengarajan *et al.* 2019; Banerjee *et al.* 2023). For assays with starved adults, young adults (∼1 day old) were washed in a watch glass and then starved on a 9 cm 2% NGM plate without bacteria for 3 h. Animals were placed within an annular ring of Whatman paper soaked in 20 mM copper chloride (CuCl_2_) solution to prevent the worms from migrating off the plate, since copper is aversive to *C. elegans* (Campbell *et al.* 2017). After 3 h of starvation, the paper ring was removed. Animals were then washed off the plate into M9 buffer and then washed twice in M9 and once in ddH_2_O in a watch glass. For testing, animals were transferred from the watch glass onto the center of a 9 cm 2% NGM plate using a piece of Whatman paper. For assays with dauer larvae, dauers were generated by transferring 8–10 young adults onto 2% NGM plates with a lawn of OP50 bacteria and leaving the plate at room temperature for 10–14 days until the bacterial lawn was consumed. Dauers were isolated from other life stages on the plate using a sodium dodecyl sulfate (SDS) resistance assay (Karp 2016) as previously described (Banerjee *et al.* 2023). The dauers were then transferred in water drops onto 2% NGM plates for assays.

CO_2_ chemotaxis assays were performed as previously described (Guillermin *et al.* 2017; Rengarajan *et al.* 2019; Banerjee *et al.* 2023). Animals were placed onto the center of a 9 cm 2% NGM plate. The CO_2_ stimulus (the test concentration of CO_2_, 21% O_2_, balance N_2_) and air stimulus (21% O_2_, balance N_2_) were pumped through holes in opposite sides of the plate lid at a rate of 2 mL/min (for adults) or 0.5 mL/min (for dauers) to generate a CO_2_ gradient (Supplementary Fig. 1a) using a syringe pump (PHD 2000, Harvard Apparatus), with the syringe output connected to the plate lid via ¼-inch flexible PVC tubing. Assay duration was 20 min (for adults) or 1 h (for dauers). After the assay, the number of animals within a 20-mm diameter circle under each gas inlet (for adults) or within 30-mm segments on the side of the plate (for dauers) were counted (Supplementary Fig. 1b and c). For transgenic strains with extrachromosomal arrays, only animals expressing the fluorescent transgene were counted. A chemotaxis index (CI) was then calculated as


$$\mathrm{CI}=\frac{\#\;\mathrm{animals}\mathrm{at}CO_{2}-\#\;\mathrm{animals}\mathrm{at}\mathrm{air}\mathrm{control}}{\#\;\mathrm{animals}\mathrm{at}CO_{2}+\#\;\mathrm{animals}\mathrm{at}\mathrm{air}\mathrm{control}}.$$

To account for directional bias due to vibration or other stimuli, assays were conducted in pairs, with the gas gradient in opposite orientations. If the absolute value of the difference in CI between the 2 assays in the pair was ≥0.9, both assays were discarded as behavior was assumed to result from directional bias. Assays were also discarded if fewer than 7 animals navigated into the combined scoring regions. For strain RB2575, which showed decreased motility, single assays within a pair that had more than 7 animals in the combined regions were scored and included in the analysis even if fewer than 7 animals moved in the other assay in the pair, provided there was no directional bias within the pair. In the case of the neuropeptide genes *ins-1*, *flp-2*, and *flp-17*, 2 independent alleles of each gene were tested in CO_2_ chemotaxis assays.

### Histamine assays with dauers

Transgenic dauers expressing the histamine-gated chloride channel HisCl1 (Pokala *et al.* 2014) were isolated using 1% SDS treatment as described above. Dauers were then incubated in 20 mM histamine solution (in dH_2_O) for 1 h prior to assays. For the no-histamine controls, dauers were incubated in dH_2_O without histamine for the same duration. Dauers were then transferred onto 2% NGM plates without bacteria, with or without 20 mM histamine, and CO_2_ chemotaxis assays were performed as described above.

### Microscopy

For starved adults, 1-day-old adults were starved for 3 h prior to imaging. Dauers for imaging were isolated by SDS treatment as described above. Animals were anesthetized using 10 mM levamisole and placed onto 2% agarose pads on glass slides. Imaging was performed with a Zeiss Axio Observer inverted wide-field fluorescent microscope equipped with a Colibri 7 for LED fluorescence illumination, a Plan-APOCHROMAT 20× objective lens, a Hamamatsu ORCA-Flash 4.0 camera, and Zen software (Zeiss). For Supplementary Fig. 3, images were captured as z-stacks and maximum intensity projections were constructed using Fiji (Schindelin *et al.* 2012).

### Statistical analysis

Statistical tests were performed using GraphPad Prism v9.3.1. Normality was determined using the D’Agostino–Pearson omnibus normality test. If data were not normally distributed, nonparametric tests were used. Power analyses were performed using G*Power v3.1.9.6 (Faul *et al.* 2007).

## Results

### Distinct sets of interneurons promote CO_2_ attraction in dauers vs starved adults

**Fig. 1.**
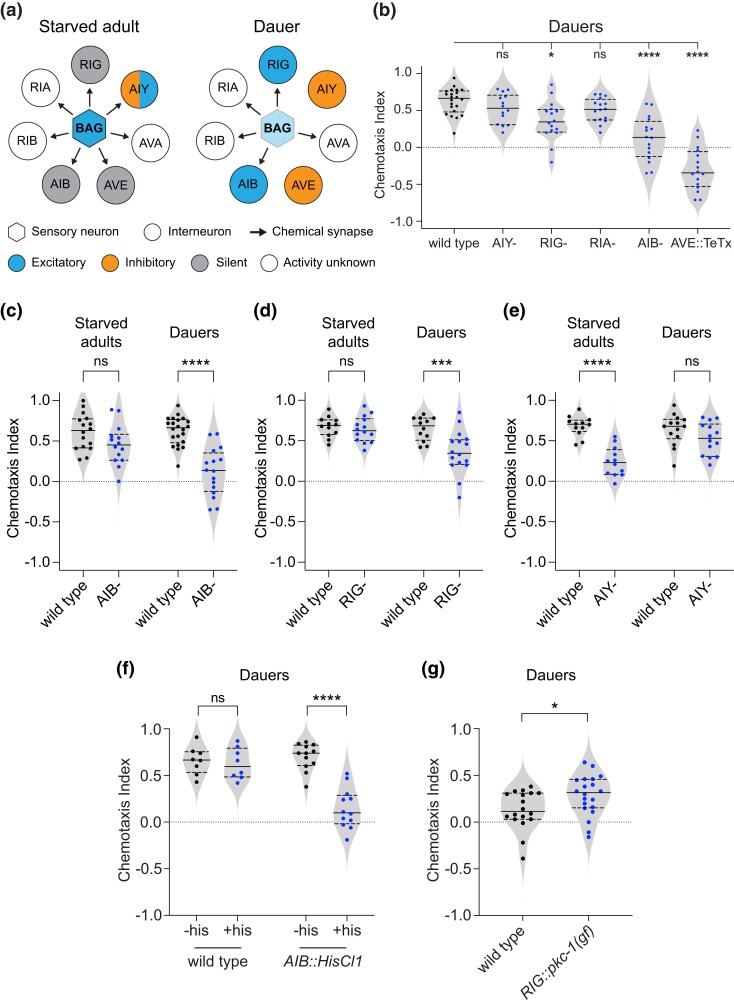
Distinct interneurons establish CO_2_ attraction in starved adults vs dauers. a) Synaptic connectivity of the BAG neurons with downstream interneurons and their CO_2_-evoked neuronal activity in adults vs dauers (White *et al.* 1986; Rengarajan *et al.* 2019; Banerjee *et al.* 2023; Yim *et al.* 2023). The lighter shade of the BAG neurons in dauers indicates a reduced CO_2_-evoked calcium response, as previously reported (Banerjee *et al.* 2023). Adapted from Banerjee *et al.* (2023). b) The interneurons RIG, AIB, and AVE promote CO_2_ attraction in dauers. Strains where specific pairs of interneurons were genetically ablated (AIY-, RIG-, RIA-, or AIB-) or silenced with tetanus toxin (AVE::TeTx) were used. ****P < 0.0001, *P < 0.05, ns = not significant, 1-way ANOVA with Dunnett's posttest. *n* = 14–22 trials per genotype. Responses shown are to 10% CO_2_. c–e) Distinct interneurons establish CO_2_ attraction in starved adults vs dauers. Behavioral responses of starved adults and dauers with genetically ablated AIB (c), RIG (d), and AIY (e) neurons in CO_2_ chemotaxis assays. ****P < 0.0001, ***P < 0.001, ns = not significant, 2-way ANOVA with Sidak's posttest. *n* = 12–22 trials per genotype. Responses shown are to 10% CO_2_. f) Behavioral responses of wild-type dauers and transgenic dauers with AIB-specific expression of the histamine-gated chloride channel HisCl1 (Pokala *et al.* 2014). Dauers expressing HisCl1 in AIB (*AIB::HisCl1*) show significantly reduced CO_2_ attraction when treated with exogenous histamine (+his) compared with untreated controls (−his), indicating that transiently silencing AIB reduces CO_2_ attraction in dauers. ****P < 0.0001, ns = not significant, 2-way ANOVA with Sidak's posttest. *n* = 8–12 assays per genotype and condition. Responses are to 10% CO_2_. g) Dauers with RIG-specific expression of a *pkc-1* gain-of-function (*gf*) allele show significantly enhanced CO_2_ attraction compared with wild-type dauers. *P < 0.05, Welch's *t*-test. *n* = 18–20 assays per genotype. Responses are to 1% CO_2_. For b–g), each data point represents a single chemotaxis assay; solid lines indicate medians and dashed lines indicate interquartile ranges.

In both starved adults and dauers, CO_2_ is detected by the paired BAG sensory neurons in the head (Hallem and Sternberg 2008; Hallem, Spencer, *et al.* 2011; Smith *et al.* 2013; Rengarajan *et al.* 2019; Banerjee *et al.* 2023). However, many of the interneurons downstream of BAG show different patterns of CO_2_-evoked neural activity in starved adults vs dauers (Fig. 1a) (Banerjee *et al.* 2023), which may in part reflect differences in synaptic connectivity of these interneurons with the BAG neurons at the two life stages (Fig. 1a) (White *et al.* 1986; Varshney *et al.* 2011; Bhattacharya *et al.* 2019; Yim *et al.* 2023). To understand the contribution of these different interneurons to CO_2_ attraction at the two life stages, we used a CO_2_ chemotaxis assay (Supplementary Fig. 1) to examine the behavioral responses of strains where individual interneurons downstream of BAG were either genetically ablated or silenced (Carrillo *et al.* 2013; Guillermin *et al.* 2017; Katz *et al.* 2018; Rengarajan *et al.* 2019). We focused on the RIG, AIY, AVE, AIB, and RIA interneurons; strains lacking RIB and AVA function were not tested because they did not produce enough dauers for chemotaxis assays, indicating a possible role for these neurons in promoting dauer entry.

We found that RIG- and AIB-ablated dauers showed reduced CO_2_ attraction compared with wild type, whereas AVE-silenced dauers were repelled by instead of attracted to CO_2_ (Fig. 1b, Supplementary Fig. 2a and b). In contrast, the AIY and RIA neurons were not required for CO_2_ attraction in dauers across all tested concentrations (Fig. 1b, Supplementary Fig. 2c and d). Thus, RIG, AIB, and AVE promote CO_2_ attraction in dauers, whereas AIY and RIA do not. We note that whereas the RIG, AIY, and AIB promoters we used to genetically ablate these interneurons showed the expected cell-specific expression pattern in starved adults and dauers, the promoter used to genetically silence AVE (*opt-3*) showed faint expression in several neurons in addition to AVE in dauers (Supplementary Fig. 3). Thus, we cannot exclude the possibility that one or more of these *opt-3*-expressing neurons also contribute to CO_2_ response in dauers.

To further investigate the roles of CO_2_ microcircuit interneurons in regulating CO_2_ attraction across life stages, we compared the behavioral responses of interneuron-ablated starved adults and dauers using a CO_2_ chemotaxis assay. We found that wild-type and AIB-ablated starved adults were similarly attracted to CO_2_, suggesting that these neurons drive CO_2_ attraction in dauers but not starved adults (Fig. 1c). RIG is also required for CO_2_ attraction in dauers but not starved adults (Fig. 1d). In contrast, AIY is required for CO_2_ attraction in starved adults (Rengarajan *et al.* 2019) but not dauers (Fig. 1e), whereas RIA is not required to establish CO_2_ attraction at either life stage (Fig. 1b) (Rengarajan *et al.* 2019). To confirm the role of AIB in regulating CO_2_ response in dauers, we examined the behavioral responses of dauers in which the AIB neurons were chemogenetically silenced using the histamine-gated chloride channel HisCl1 (Pokala *et al.* 2014). Dauers expressing HisCl1 specifically in AIB showed a significant reduction in CO_2_ attraction when treated with exogenous histamine compared with untreated control dauers (Fig. 1f), confirming that AIB promotes CO_2_ attraction in dauers. To further confirm the role of RIG in promoting CO_2_ attraction in dauers, we hyperactivated RIG by expressing a gain-of-function allele of the protein kinase C (*pkc-1*) gene (Sieburth *et al.* 2005; Sieburth *et al.* 2007) specifically in RIG (Guillermin *et al.* 2017). We found that hyperactivation of RIG results in enhanced CO_2_ attraction in dauers (Fig. 1g), providing additional evidence that RIG promotes CO_2_ attraction in dauers. Thus, both AIB and RIG promote CO_2_ attraction in dauers but not starved adults. Together, our results demonstrate that distinct interneurons are required for establishing the same valence state at 2 different life stages.

**Fig. 2.**
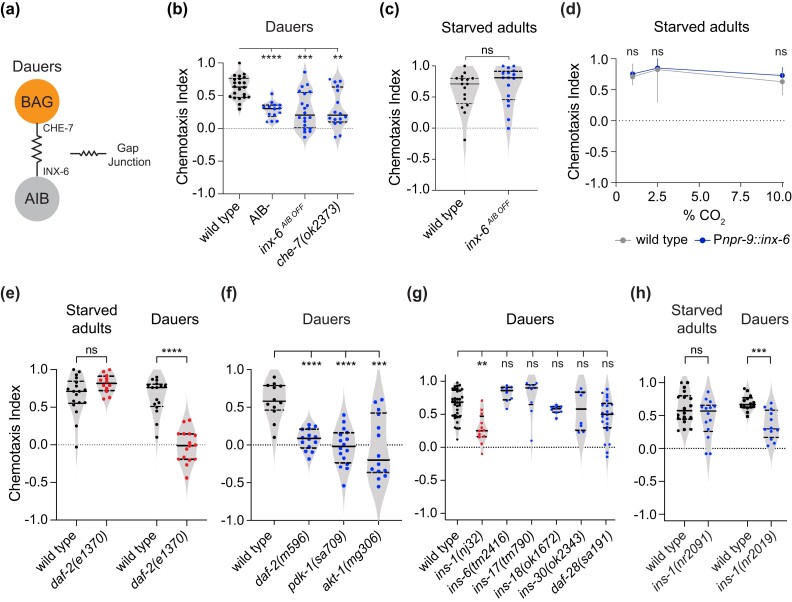
A BAG-AIB gap junction complex and insulin signaling are required for CO_2_ attraction in dauers but not starved adults. a) A gap junction complex is formed between the BAG sensory neurons and the AIB interneurons in dauers by selective expression of the innexin subunit INX-6 in AIB (Bhattacharya *et al.* 2019). INX-6 partners with the innexin subunit CHE-7, which is expressed in BAG, to form gap junctions (Bhattacharya *et al.* 2019). b) Behavioral responses of AIB-ablated (AIB-) dauers, dauers containing an AIB-specific knockout of *inx-6* (*inx-6^AIB OFF^*) (Bhattacharya *et al.* 2019), and *che-7* mutant dauers. ****P < 0.0001, ***P < 0.001, **P < 0.01, 1-way ANOVA with Dunnett's posttest. *n* = 14–22 trials per genotype. c) CO_2_ attraction in starved adults remains unaffected in *inx-6* (*inx-6^AIB OFF^*) mutants. ns = not significant, Mann–Whitney test. *n* = 16 trials per genotype. d) Ectopic expression of INX-6 in AIB does not alter CO_2_ responses in starved adults. Behavioral responses of starved adults expressing *inx-6* specifically in AIB under the control of the *npr-9* promoter (P*npr-9*::*inx-6*) to CO_2_ across concentrations. *n* = 12–16 trials per genotype and condition. Lines show medians and interquartile ranges. ns = not significant, 2-way ANOVA with Sidak's posttest. e) Behavioral responses of wild-type vs *daf-2* mutant starved adults and dauers. DAF-2 promotes CO_2_ attraction in dauers but not starved adults. *n* = 12–18 trials per life stage and genotype. ****P < 0.0001, ns = not significant, 2-way ANOVA with Sidak's posttest. f) Behavioral responses of *daf-2*, *pdk-1*, and *akt-1* mutant dauers. *n* = 12–16 trials per genotype. ****P < 0.0001, ***P < 0.001, 1-way ANOVA with Dunnett's posttest. g) Behavioral responses of dauers carrying loss-of-function mutations in candidate ILP genes. **P < 0.01, ns = not significant, Kruskal–Wallis test with Dunn's posttest. *n* = 8–34 trials per genotype. h) Behavioral responses of starved adults and dauers containing a loss-of-function mutation in the insulin-like peptide gene *ins-1*. INS-1 promotes CO_2_ attraction in dauers but not starved adults. ***P < 0.001, ns = not significant, 2-way ANOVA with Sidak's posttest. *n* = 12–16 trials per life stage and genotype. For b–c) and e–h), each data point represents a single chemotaxis assay; solid lines indicate medians and dashed lines indicate interquartile ranges. Responses shown are to 10% CO_2_.

We then tested the effects of dauer-specific changes in neuronal connectivity on the life stage–specific requirement for interneurons in establishing CO_2_ attraction. AIB was previously shown to form gap junctions with BAG in dauers but not adults—the innexin INX-6 is expressed in AIB specifically in dauers, where it forms a gap junction complex with CHE-7, a gap junction subunit expressed in BAG (Fig. 2a) (Bhattacharya *et al.* 2019). Consistent with previous results (Bhattacharya *et al.* 2019), *che-7* mutant dauers, as well as dauers where *inx-6* expression is eliminated specifically in AIB (*inx-6^AIB OFF^*), showed significantly reduced CO_2_ attraction compared with wild-type dauers (Fig. 2b). In contrast to *inx-6*^*AIB OFF*^ dauers, *inx-6*^*AIB OFF*^ starved adults showed no defects in CO_2_ attraction (Fig. 2c). Further, ectopic expression of *inx-6* in AIB did not affect CO_2_ responses in starved adults (Fig. 2d), indicating that additional mechanistic differences underlie CO_2_ attraction in dauers vs starved adults.

### Insulin signaling promotes CO_2_ attraction in dauers but not starved adults

We then investigated the molecular mechanisms that regulate CO_2_ attraction in dauers and starved adults. Insulin signaling regulates entry into the dauer state (Hu 2007) as well as a diverse array of chemosensory behaviors in adults (Tomioka *et al.* 2006; Hallem and Sternberg 2008; Adachi *et al.* 2010). Moreover, we previously showed that insulin signaling modulates the CO_2_-evoked neuronal activity of AIB in dauers (Banerjee *et al.* 2023). We therefore examined the role of insulin signaling in regulating CO_2_ attraction in starved adults and dauers.

We first examined the CO_2_ response of animals carrying a loss-of-function mutation in the *daf-2* gene, which encodes the sole *C. elegans* insulin receptor (Hu 2007). We found that loss of *daf-2* function had no effect on CO_2_ attraction in starved adults (Fig. 2e). In contrast, *daf-2* mutant dauers were unresponsive to CO_2_, suggesting that *daf-2* promotes CO_2_ attraction in dauers (Fig. 2e–f). The *daf-2* gene is broadly expressed in multiple tissues in both adults and dauers (Murphy and Hu 2013; Martinez *et al.* 2020). To identify the tissues where *daf-2* might function to regulate CO_2_ attraction in dauers, we performed CO_2_ chemotaxis assays on *daf-2* mutant dauers in which DAF-2 function was specifically restored in either neurons, intestine, or muscle. Restoring DAF-2 function in neurons, but not intestine or muscle, partially yet significantly rescued the chemotaxis defect of *daf-2* mutant dauers (Supplementary Fig. 4). These results suggest that DAF-2 primarily functions in neurons to mediate CO_2_ attraction in dauers. In addition, we found that dauers carrying loss-of-function mutations in the 3-phosphoinositide-dependent-kinase-1 gene *pdk-1* and the protein kinase B (Akt/PKB) gene *akt-1*, both of which act downstream of *daf-2* (Hu 2007), showed significantly reduced CO_2_ attraction (Fig. 2f). Thus, insulin signaling promotes CO_2_ attraction in dauers but not starved adults.

We next sought to identify the DAF-2 ligands that modulate CO_2_ response. The *C. elegans* genome encodes 40 insulin-like peptides (ILPs) (Li and Kim 2008). To identify ILPs that may be involved in regulating CO_2_ response in dauers, we examined a subset of ILP genes known to be transcriptionally upregulated upon dauer entry (Lee *et al.* 2017). We found that dauers carrying two independent loss-of-function mutations in the *ins-1* gene showed significantly reduced CO_2_ attraction compared with wild-type dauers (Fig. 2g and h). In contrast, CO_2_ attraction in starved adults was unaffected in *ins-1* mutants (Fig. 2h). Thus, the modulatory effects of insulin signaling on CO_2_ attraction in dauers are mediated at least in part by INS-1 peptides. Together, our results demonstrate that insulin signaling regulates CO_2_ response valence in a life stage–dependent manner.

### Both shared and life stage–specific neuropeptides and neurotransmitters regulate CO_2_ attraction

**Fig. 3.**
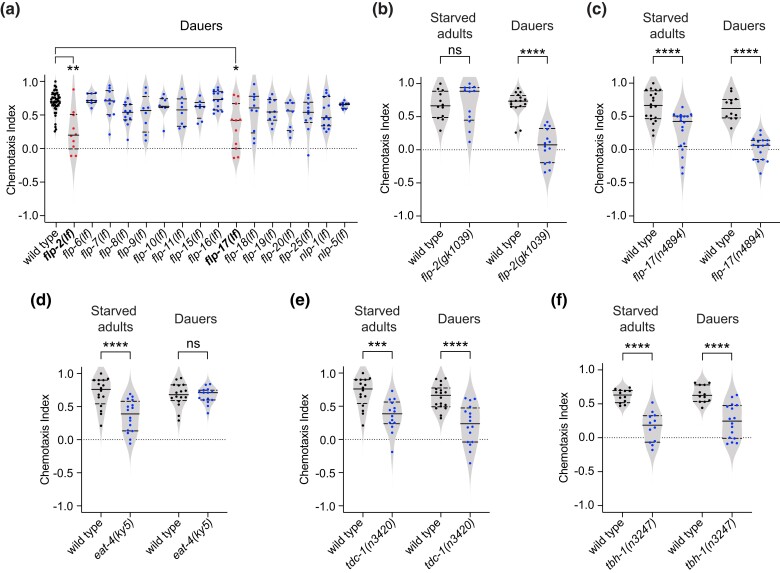
Both shared and life stage–specific neurotransmitters and neuropeptides shape CO_2_ attraction in starved adults vs dauers. a) A reverse genetic screen for neuropeptides that regulate CO_2_ attraction in dauers. Behavioral responses of dauers with loss-of-function mutations in neuropeptide genes. **P < 0.01, *P < 0.05, Kruskal–Wallis test with Dunn's posttest. *n* = 8–42 trials per genotype. The specific loss-of-function (*lf*) alleles used for *flp-2* and *flp-17* were *ok3351* and *ok3587*, respectively. Loss-of-function (*lf*) alleles used for other neuropeptide genes are indicated in the Materials and methods and Supplementary Table 1. b) and c) Behavioral responses of starved adults and dauers containing loss-of-function mutations in the neuropeptide genes *flp-2* (b) and *flp-17* (c). ****P < 0.0001, ns = not significant, 2-way ANOVA with Sidak's posttest. *n* = 12–20 trials per genotype and life stage. d–f) Behavioral responses of starved adults and dauers containing loss-of-function mutations in the vesicular glutamate transporter gene *eat-4* (d), the tyrosine decarboxylase gene *tdc-1* (e), and the tyramine β-hydroxylase gene *tbh-1* (f). ****P < 0.0001, ***P < 0.001, ns = not significant, 2-way ANOVA with Sidak's posttest. *n* = 12–18 trials per genotype and life stage. For a–f), each data point represents a single chemotaxis assay; solid lines indicate medians and dashed lines indicate interquartile ranges. Responses shown are to 10% CO_2_.

Neuropeptide signaling plays a critical role in modulating neuronal function and behavior across species (Li and Kim 2008) and was previously shown to modulate the CO_2_-evoked behavioral responses of *C. elegans* adults (Guillermin *et al.* 2017; Rengarajan *et al.* 2019). To examine whether CO_2_ responses in dauers are regulated by neuropeptides, we performed a candidate screen of dauers carrying loss-of-function mutations in 16 neuropeptide genes (Fig. 3a). We focused on neuropeptide genes that are either expressed in BAG (Hallem, Spencer, *et al.* 2011), transcriptionally upregulated upon dauer entry (Lee *et al.* 2017), known to regulate CO_2_ response in adults (Guillermin *et al.* 2017; Rengarajan *et al.* 2019), or known to encode neuropeptides that are dependent on the neuropeptide-processing gene *sbt-1*, since *sbt-1* promotes dauer entry and regulates CO_2_ attraction in dauers (Lee *et al.* 2017).

We found that the FMRFamide-like neuropeptide gene *flp-16* and the neuropeptide-like protein gene *nlp-1*, which were previously shown to regulate CO_2_ attraction in starved adults (Rengarajan *et al.* 2019), are not required to promote CO_2_ attraction in dauers (Fig. 3a). Thus, NLP-1 and FLP-16 neuropeptides play adult-specific roles in regulating CO_2_ response. However, dauers lacking the FMRFamide-like neuropeptide gene *flp-2* showed significantly reduced CO_2_ attraction (Fig. 3a and b), whereas starved adults lacking *flp-2* responded normally to CO_2_ (Fig. 3b). Thus, FLP-2 neuropeptides are required to establish CO_2_ attraction in dauers but not starved adults. In addition, we found that both dauers and starved adults carrying loss-of-function mutations in the BAG-expressed neuropeptide gene *flp-17* showed significantly reduced CO_2_ attraction (Fig. 3a and c). Thus, our results demonstrate that FLP-17 promotes CO_2_ attraction in both dauers and starved adults.

We also investigated the role of neurotransmitters in regulating CO_2_ attraction in starved adults vs dauers. We first examined the role of glutamate signaling, since the CO_2_-detecting BAG neurons express the vesicular glutamate transporter EAT-4 (Serrano-Saiz *et al.* 2013) and well-fed adults carrying a loss-of-function mutation in the *eat-4* gene show dramatically reduced CO_2_ repulsion (Guillermin *et al.* 2017). We found that starved adults lacking *eat-4* showed significantly reduced CO_2_ attraction (Fig. 3d). In contrast, CO_2_ attraction remained unaffected in *eat-4* mutant dauers (Fig. 3d). Thus, glutamate signaling is required for CO_2_ attraction in starved adults but not dauers. Finally, we examined the role of biogenic amines in regulating CO_2_ attraction. Both the tyrosine decarboxylase gene *tdc-1*, which is required for the biosynthesis of tyramine and octopamine (Chase and Koelle 2007), and the tyramine β-hydroxylase gene *tbh-1*, which is required for the conversion of tyramine to octopamine (Chase and Koelle 2007), promote CO_2_ attraction in starved adults (Fig. 3e and f) (Rengarajan *et al.* 2019). We found that both *tdc-1* and *tbh-1* also promote CO_2_ attraction in dauers, since *tdc-1* and *tbh-1* mutant dauers show decreased CO_2_ attraction (Fig. 3e and f). Thus, while glutamate signaling regulates CO_2_ attraction specifically in starved adults, biogenic amine signaling regulates CO_2_ attraction in both starved adults and dauers. Together, our results identify both shared and life stage–specific neuropeptides and neurotransmitters that regulate CO_2_ attraction in starved adults vs dauers, suggesting that distinct combinations of neuropeptides and neurotransmitters regulate CO_2_ attraction at the two life stages.

## Discussion

**Fig. 4.**
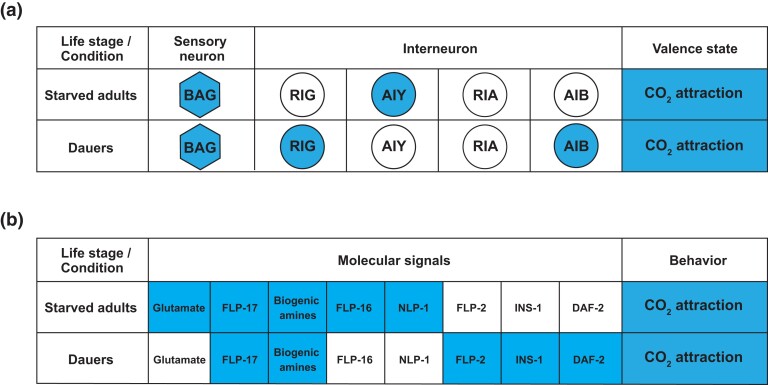
Distinct mechanisms shape CO_2_ response valence across life stages. a) Table showing the requirement for the BAG sensory neurons and downstream interneurons in establishing CO_2_ response valence in starved adults vs dauers. Neurons involved in establishing CO_2_ attraction are shaded; neurons not required for CO_2_ attraction are unshaded. b) Table showing the requirement for different molecular signals in establishing CO_2_ response valence across life stages. Neurotransmitters and neuropeptides required to establish CO_2_ attraction are indicated by shaded boxes; the lack of requirement for a particular molecular signal in establishing CO_2_ attraction is indicated by an unshaded box.

We have shown that the same valence state, CO_2_ attraction, is established in dauers and starved adults by distinct cellular and molecular mechanisms. The same CO_2_-detecting sensory neurons are required for CO_2_ attraction in both dauers and starved adults, but distinct interneurons mediate CO_2_ attraction at the two life stages (Fig. 4a) (Hallem, Dillman, *et al.* 2011a; Rengarajan *et al.* 2019; Banerjee *et al.* 2023). The requirement for different interneurons at the dauer vs adult stage may arise from anatomical as well as functional differences in neuronal connectivity at the two life stages. For example, the AIY neurons promote CO_2_ attraction in starved adults but not dauers (Fig. 4a), consistent with the dauer-specific loss of chemical synapses between BAG and AIY (Yim *et al.* 2023). However, AIY still displays CO_2_-evoked activity in dauers (Banerjee *et al.* 2023), suggesting that this activity is independent of BAG-AIY chemical synapses. In addition, the AIB and RIG neurons promote CO_2_ attraction in dauers but not starved adults despite forming chemical synapses with the BAG neurons at both life stages (Fig. 1a) (White *et al.* 1986; Yim *et al.* 2023). The distinct requirement for AIB in mediating dauer CO_2_ attraction arises due to dauer-specific BAG-AIB gap junctions (Bhattacharya *et al.* 2019). The role of RIG in selectively promoting CO_2_ attraction in dauers may arise from changes in functional connectivity between BAG and RIG, although differences in anatomical connectivity between RIG and other neurons may also contribute. For example, RIG receives dauer-specific synaptic input from the IL2 neurons, which are required for nictation, a dauer-specific behavior that promotes dispersal by facilitating phoretic interactions between dauers and larger invertebrates (Lee *et al.* 2012) (Yim *et al.* 2023). Our results raise the possibility that RIG activity drives CO_2_ attraction specifically in dauers to enable dauers to coordinate CO_2_ response with nictation.

Our findings also demonstrate that distinct combinations of neurotransmitters and neuropeptides are required for CO_2_ attraction at the two life stages (Fig. 4b). Further studies of the effects of these molecular signals on CO_2_ microcircuit function will reveal the regulatory principles that enable the worm to establish the same valence state under distinct physiological conditions. For instance, biogenic amine signaling promotes CO_2_ attraction in starved adults by regulating the CO_2_-evoked activity of AIY (Rengarajan *et al.* 2019). In dauers, biogenic amine signaling is also required for CO_2_ attraction despite the lack of requirement for AIY (Fig. 4b), suggesting that biogenic amine signaling promotes CO_2_ attraction via distinct neural mechanisms at the two life stages. Taken together, our results demonstrate that different mechanisms may operate at different life stages to promote the same valence state. In future studies, it will be interesting to explore whether life stage–specific chemosensory mechanisms function to couple chemosensory responses to other life stage–specific, ethologically relevant behavioral programs.

## Supplementary Material

jkad271_Supplementary_Data

## Data Availability

Strains and plasmids are available upon request. All raw data and statistical analysis from this study are included in Supplementary File S1. Supplemental material available at G3 online.
