# Supplementary material for: Distinct neurogenetic mechanisms establish the same chemosensory valence state at different life stages in Caenorhabditis elegans
Source: G3 (Bethesda). 2023 Nov 23;14(2):jkad271. doi: 10.1093/g3journal/jkad271 (PMC10849362; doi:10.1093/g3journal/jkad271)
Supplement: jkad271_Supplementary_Data [file jkad271_supplementary_data.zip › Supplemental_Figure_Legends_G3-2023-404700.pdf]

Supplemental Figure Legends

Fig. S1. CO_2 chemotaxis assays for *C. elegans* adults and dauers. A. Schematic of the CO_2 chemotaxis assay. Animals were placed in the center of a 90 mm NGM agar plate. A CO_2 gradient was established by delivering the specified gas mixtures through holes in either side of the plate lid. Animals were allowed to navigate in the CO_2 gradient (indicated by the shaded rectangle below). At the end of each assay, animals on each side of the plate were counted and results were scored as a chemotaxis index (CI) according to the formula indicated. Adapted from Guillermin *et al.,* 2017 (GUILLERMIN *et al.* 2017). **B.** For adult assays, the number of animals within a 20 mm diameter circle centered under each gas inlet was counted. **C**. For dauer assays, the number of animals within the indicated 30 mm segments on both sides of the plate was counted. For B-C, dashed red lines indicate the area within which animals were placed at the beginning of the assay.

Fig. S2. Behavioral responses of dauers with genetically ablated AIY, RIG, RIA, and AIB neurons to CO_2 across concentrations. Graphs show medians and interquartile ranges. n = 12-22 trials per genotype and life stage. ****p<0.0001, ***p<0.001, **p<0.01, *p<0.05, ns = not significant, two-way ANOVA test with Sidak's post-test.

Fig. S3. The RIG-, AIY- and AIB-specific promoters show similar expression patterns in starved adults and dauers. Epifluorescent images of transgenic starved adults and dauers expressing yellow cameleon YC3.60 in RIG (P*twk-3::YC3.60*), AIY (P*ttx-3::YC3.60*), or AVE (P*opt-3::YC3.60*); or TagRFP in AIB (P*npr-9*::*TagRFP*). Arrowheads indicate expression of the *opt-3* promoter in additional neurons in dauers. The bright intestinal fluorescence in *Pnpr-9*::*TagRFP* starved adults is the result of autofluorescence. Scale bars indicate 25 µm. The *Pnpr-9::TagRFP* image is from Banerjee *et al.,* 2023 (BANERJEE *et al.* 2023).

Fig. S4. DAF-2 functions in neurons to regulate CO_2 attraction in dauers. Restoring DAF-2 function in neurons partially rescues the chemotaxis defect of *daf-2* mutant dauers. Behavioral responses of transgenic *daf-2* mutant dauers where *daf-2* function is restored in neurons (P*rgef-1*::*daf-2*), intestine (P*ges-1*::*daf-2*), or muscle (P*myo-3*::*daf-2*). n = 18-24 trials per genotype. ****p<0.0001, ***p<0.001, ns = not significant, one-way ANOVA with Dunnett's post-test. Each data point indicates a single chemotaxis assay. Solid lines in violin plot show medians and dashed lines show interquartile ranges. Responses shown are to 10% CO_2.
