# Supplementary material for: Distinct neurogenetic mechanisms establish the same chemosensory valence state at different life stages in Caenorhabditis elegans
Source: G3 (Bethesda). 2023 Nov 23;14(2):jkad271. doi: 10.1093/g3journal/jkad271 (PMC10849362; doi:10.1093/g3journal/jkad271)
Supplement: jkad271_Supplementary_Data [file jkad271_supplementary_data.zip › Banerjee et al Table S1 2023-11-29.pdf]

**Table S1.** The list of strains used. *lf* = loss-of-function mutation; *ts* = temperature-sensitive.

| Strain | Genotype | Strain description | Source and References |
|---|---|---|---|
| N2 | Wild-type Bristol | wild type | CGC |
| EAH284 | *bruEx138[ttx-3::casp-3(p17); ttx-3::casp-3(p12); myo-2::dsRed]* | AIY ablation | Hallem lab[1,2] |
| EAH268 | *bruEx160[twk-3::casp-3(p17); twk-3::casp-3(p12); myo-2::dsRed]* | RIG ablation | Hallem lab[1,2] |
| EAH 202 | *peIs578[npr-9::casp1, npr-9::Venus, unc-122::mCherry]* | AIB ablation | Iino lab[3] |
| EAH319 | *bruEx171[glr-3::casp-3(p17); glr-3::casp-3(p12); myo-2::dsRed]* | RIA ablation | Hallem lab[1,2] |
| OS4977 | *nsEx2847[pept-3::TeTx, elt-2::mCherry]* | Tetanus toxin in AVE | Shaham lab[4] |
| PS6028 | *syEx1134[twk-3::YC3.60; pax-2::GFP]* | Cameleon in RIG | Sternberg lab[1] |
| IK1405 | *njEx568[ttx-3::YC3.60, ges-1::NLS-RFP]* | Cameleon in AIY | Mori lab[1] |
| OH13918 | *otIs643[npr-9::*TagRFP*]* | TagRFP in AIB | Hobert lab |
| PS5932 | *lin-15(n765); syEx1111[opt-3::YC3.60, lin-15(+)]* | Cameleon in AVE | Sternberg lab |
| CX15457 | *kyIs620[inx-1::HisCl1::SL2::GFP, myo-3::mCherry]* | HisCl1 in AIB | Bargmann lab[5] |
| EAH287 | *bruEx163[twk-3::pkc-1(gf)::SL2::GFP]* | *pkc-1(gf)* in RIG | Hallem lab[1] |
| RB1834 | *che-7(ok2373)* | *che-7(lf)* mutant | CGC[6] |
| OH14014 | *inx-6(ot804[inx-6::SL2::NLS::YFP::H2B];ot840[inx-6Del > TAATTA::SL2::NLS::YFP::H2B])* | Dauer-specific expression of *inx-6* in AIB is eliminated | Hobert lab[7] |
| OH14529 | *otTi19(Si[Pnpr-9::INX-6::GFP])* | *inx-6* expression in AIB in starved adults | Hobert lab[7] |
| CB1370 | *daf-2(e1370)* | *daf-2(lf)* mutant (*ts* allele) | CGC[8] |
| DR1565 | *daf-2(m596)* | *daf-2(lf)* mutant (*ts* allele) | CGC[9] |
| EAH404 | *daf-2(e1370);bruEx206[Prgef-1::daf-2]* | Pan-neuronal rescue of *daf-2* | Hallem lab (this paper) |
| EAH407 | *daf-2(e1370);bruEx209[Pges-1::daf-2]* | Intestinal rescue of *daf-2* | Hallem lab (this paper) |
| EAH408 | *daf-2(e1370);bruEx210[Pmyo-3::daf-2]* | Muscle-specific rescue of *daf-2* | Hallem lab (this paper) |
| JT709 | *pdk-1(sa709)* | *pdk-1(lf)* mutant | CGC[10] |
| BQ1 | *akt-1(mg306)* | *akt-1(lf)* mutant | CGC[11] |
| IK581 | *ins-1(nj32)* | *ins-1(lf)* mutant | Mori lab[12] |
| NS3242 | *ins-1(nr2091)* | *ins-1(lf)* mutant | Ruvkun lab[13] |
| RB1809 | *ins-30(ok2343)* | *ins-30(lf)* mutant | CGC[6] |
| VC1218 | *ins-18(ok1672)* | *ins-18(lf)* mutant | CGC[6] |
| FX2416 | *ins-6(tm2416)* | *ins-6(lf)* mutant | National BioResource Project (Mitani lab) |
| FX790 | *ins-17(tm790)* | *ins-17(lf)* mutant | National BioResource Project (Mitani lab) |
| JT191 | *daf-28(sa191)* | *daf-28(lf)* mutant (*ts* allele) | CGC[14] |
| VC2591 | *flp-2(ok3351)* | *flp-2(lf)* mutant | CGC[6] |
| VC2490 | W07E11.1 & *flp-2(gk1039)* | *flp-2(lf)* mutant | CGC[6] |

| | | | |
|---|---|---|---|
| VC2324 | *flp-6(ok3056)* | *flp-6(lf)* mutant | CGC[6] |
| RB1990 | *flp-7(ok2625)* | *flp-7(lf)* mutant | CGC[6] |
| PT501 | *flp-8(pk360)* | *flp-8(lf)* mutant | CGC[15] |
| RB2067 | *flp-9(ok2730)* | *flp-9(lf)* mutant | CGC[6] |
| PT502 | *flp-10(pk367)* | *flp-10(lf)* mutant | Barr lab[16] |
| FX2706 | *flp-11(tm2706)* | *flp-11(lf)* mutant | National BioResource Project (Mitani lab) |
| VC2504 | *flp-15(gk1186)* | *flp-15(lf)* mutant | CGC[6] |
| FX5158 | *flp-16(tm5158)* | *flp-16(lf)* mutant | National BioResource Project (Mitani lab)[1,2] |
| RB2575 | *flp-17(ok3587)* | *flp-17(lf)* mutant | CGC[6] |
| MT15933 | *flp-17(n4894)* | *flp-17(lf)* mutant | CGC[1,17] |
| VC2016 | *flp-18(gk3063)* | *flp-18(lf)* mutant | CGC[6] |
| RB1902 | *flp-19(ok2460)* | *flp-19(lf)* mutant | CGC[6,16] |
| RB2188 | *flp-20(ok2964)* | *flp-20(lf)* mutant | CGC[6,18] |
| VC1982 | *flp-25(gk1016)* | *flp-25(lf)* mutant | CGC[6,18] |
| RB1341 | *nlp-1(ok1470)* | *nlp-1(lf)* mutant | CGC[1,2,6] |
| RB1609 | *nlp-5(ok1981)* | *nlp-5(lf)* mutant | CGC[6] |
| MT6308 | *eat-4(ky5)* | *eat-4(lf)* mutant | CGC[1] |
| MT10661 | *tdc-1(n3420)* | *tdc-1(lf)* mutant | CGC[2] |
| MT9455 | *tbh-1(n3247)* | *tbh-1(lf)* mutant | CGC[19] |

**References:**


1 Guillermin, M. L., Carrillo, M. A. & Hallem, E. A. A single set of interneurons drives opposite behaviors in *C. elegans*. *Curr Biol* **27**, 2630-2639 (2017).

2 Rengarajan, S., Yankura, K. A., Guillermin, M. L., Fung, W. & Hallem, E. A. Feeding state sculpts a circuit for sensory valence in *Caenorhabditis elegans*. *Proc Natl Acad Sci USA* **116**, 1776-1781 (2019).

3 Kunitomo, H. *et al.* Concentration memory-dependent synaptic plasticity of a taste circuit regulates salt concentration chemotaxis in *Caenorhabditis elegans*. *Nat Commun* **4**, 2210 (2013).

4 Katz, M., Corson, F., Iwanir, S., Biron, D. & Shaham, S. Glia modulate a neuronal circuit for locomotion suppression during sleep in *C. elegans*. *Cell Rep* **22**, 2575-2583 (2018).

5 Pokala, N., Liu, Q., Gordus, A. & Bargmann, C. I. Inducible and titratable silencing of *Caenorhabditis elegans* neurons *in vivo* with histamine-gated chloride channels. *Proc Natl Acad Sci USA* **111**, 2770-2775 (2014).

6 *C. elegans* Deletion Mutant Consortium. Large-scale screening for targeted knockouts in the *Caenorhabditis elegans* genome. *G3* **2**, 1415-1425 (2012).

7 Bhattacharya, A., Aghayeva, U., Berghoff, E. G. & Hobert, O. Plasticity of the electrical connectome of *C. elegans*. *Cell* **176**, 1174-1189 (2019).

8 Riddle, D. L. A genetic pathway for dauer larva formation in *C. elegans. Stadler Genetics Symposium* **9**, 101-120 (1977).

9 Gems, D. *et al.* Two pleiotropic classes of *daf-2* mutation affect larval arrest, adult behavior, reproduction and longevity in *Caenorhabditis elegans*. *Genetics* **150**, 129-155 (1998).

10 Paradis, S., Ailion, M., Toker, A., Thomas, J. H. & Ruvkun, G. A PDK1 homolog is necessary and sufficient to transduce AGE-1 PI3 kinase signals that regulate diapause in *Caenorhabditis elegans*. *Genes Dev* **13**, 1438-1452 (1999).

11 Shmookler Reis, R. J., Ayyadevara, S., Crow, W. A., Lee, T. & Delongchamp, R. R. Gene categories differentially expressed in *C. elegans age-1* mutants of extraordinary longevity: new insights from novel data-mining procedures. *J Gerontol A Biol Sci Med Sci* **67**, 366-375 (2012).

12 Kodama, E. *et al.* Insulin-like signaling and the neural circuit for integrative behavior in *C. elegans*. *Genes Dev* **20**, 2955-2960 (2006).

13 Pierce, S. B. *et al.* Regulation of DAF-2 receptor signaling by human insulin and *ins-1*, a member of the unusually large and diverse *C. elegans* insulin gene family. *Genes Dev* **15**, 672-686 (2001).

14 Birnby, D. A. *et al.* A transmembrane guanylyl cyclase (DAF-11) and Hsp90 (DAF-21) regulate a common set of chemosensory behaviors in *Caenorhabditis elegans*. *Genetics* **155**, 85-104 (2000).

15 Liu, T., Kim, K., Li, C. & Barr, M. M. FMRFamide-like neuropeptides and mechanosensory touch receptor neurons regulate male sexual turning behavior in Caenorhabditis elegans. *J Neurosci* **27**, 7174-7182, doi:27/27/7174 (2007).

16 Carrillo, M. A., Guillermin, M. L., Rengarajan, S., Okubo, R. & Hallem, E. A. $O_2$-sensing neurons control $CO_2$ response in *C. elegans*. *J Neurosci* **33**, 9675-9683 (2013).

17 Horowitz, L. B., Brandt, J. P. & Ringstad, N. Repression of an activity-dependent autocrine insulin signal is required for sensory neuron development in *C. elegans*. *Development* **146**, dev182873 (2019).

18 Lee, J. S. *et al.* FMRFamide-like peptides expand the behavioral repertoire of a densely connected nervous system. *Proc Natl Acad Sci USA* **114**, E10726-E10735 (2017).

19 Alkema, M. J., Hunter-Ensor, M., Ringstad, N. & Horvitz, H. R. Tyramine functions independently of octopamine in the *Caenorhabditis elegans* nervous system. *Neuron* **46**, 247-260 (2005).
