# Supplementary material for: Distinct neurogenetic mechanisms establish the same chemosensory valence state at different life stages in Caenorhabditis elegans
Source: G3 (Bethesda). 2023 Nov 23;14(2):jkad271. doi: 10.1093/g3journal/jkad271 (PMC10849362; doi:10.1093/g3journal/jkad271)
Supplement: jkad271_Supplementary_Data [file jkad271_supplementary_data.zip › Figure_S1_G3-2023-404700.pdf]

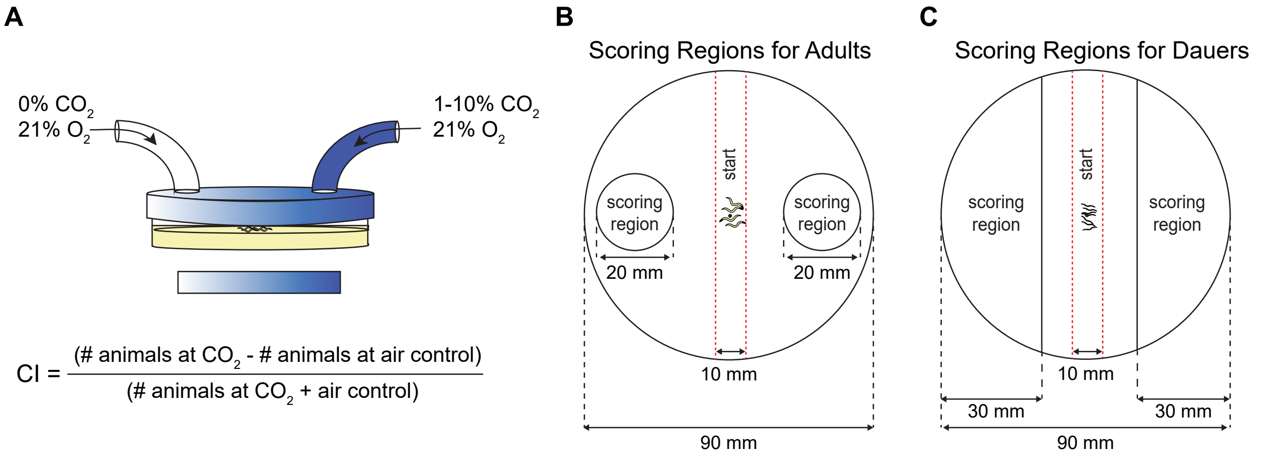
A
0% CO2
21% O2
1-10% CO2
21% O2
CI = (# animals at CO2 - # animals at air control) / (# animals at CO2 + air control)
B
Scoring Regions for Adults
start
scoring region
scoring region
20 mm
20 mm
10 mm
90 mm
C
Scoring Regions for Dauers
start
scoring region
scoring region
10 mm
30 mm
30 mm
90 mm

Figure S1
