# Supplementary figures and images for: Distinct neurogenetic mechanisms establish the same chemosensory valence state at different life stages in Caenorhabditis elegans
Source: G3 (Bethesda). 2023 Nov 23;14(2):jkad271. doi: 10.1093/g3journal/jkad271 (PMC10849362; doi:10.1093/g3journal/jkad271)

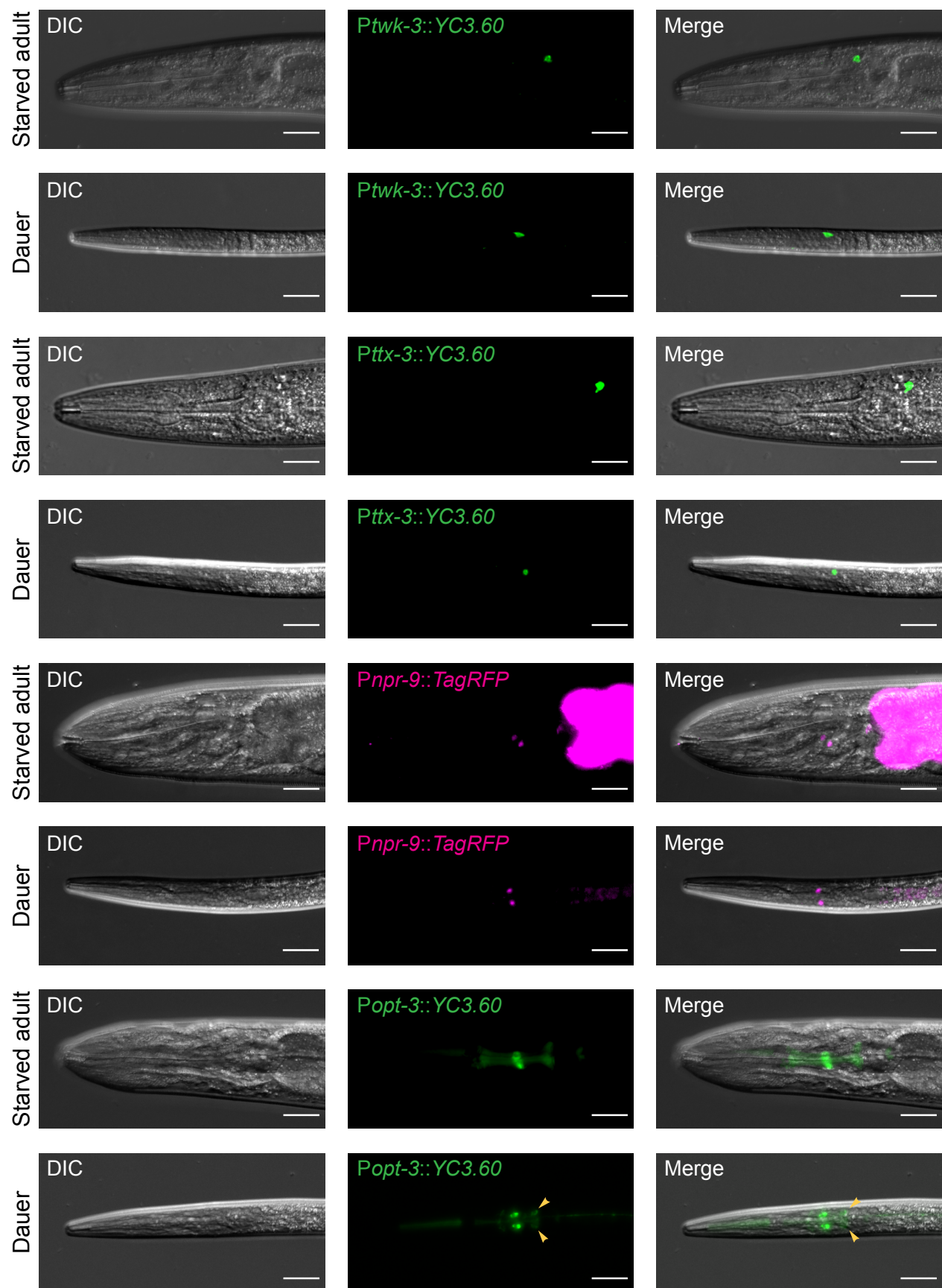

Figure S3

Supplement: jkad271_Supplementary_Data [file jkad271_supplementary_data.zip › Figure_S3_G3-2023-404700.pdf]

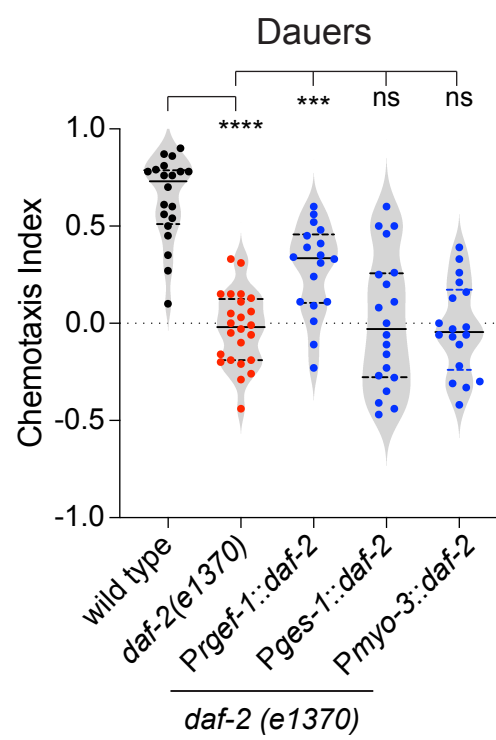

Figure S4

Supplement: jkad271_Supplementary_Data [file jkad271_supplementary_data.zip › Figure_S4_G3-2023-404700.pdf]

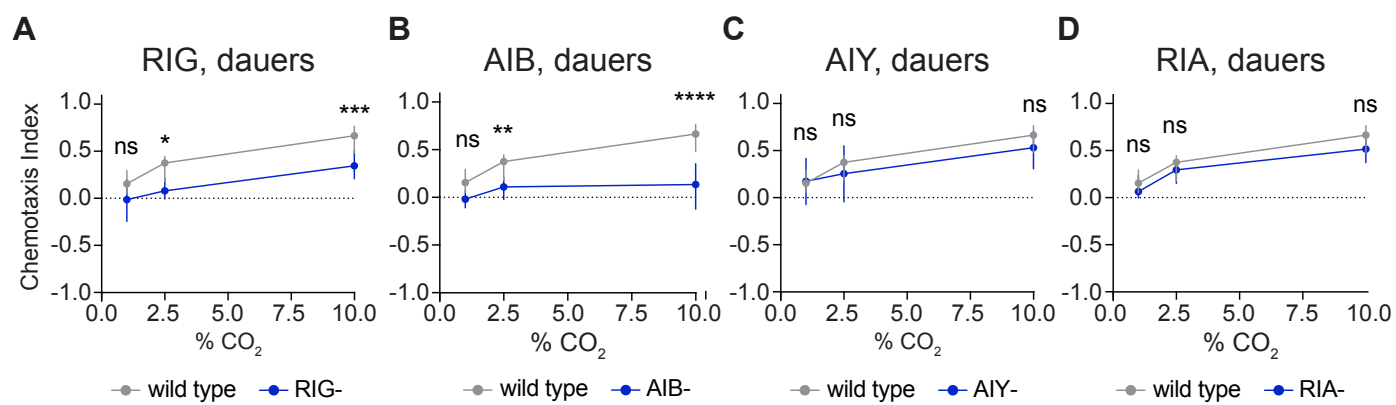

Figure S2

Supplement: jkad271_Supplementary_Data [file jkad271_supplementary_data.zip › Figure_S2_G3-2023-404700.pdf]
